# Identification and function of long non-coding RNA

**DOI:** 10.3389/fncel.2013.00168

**Published:** 2013-10-02

**Authors:** Carl Ernst, Cynthia C. Morton

**Affiliations:** ^1^Douglas Hospital Research InstituteMontreal, QC, Canada; ^2^Department of Psychiatry, McGill UniversityMontreal, QC, Canada; ^3^ Department of Obstetrics, Gynecology and Reproductive Biology, Brigham and Women’s Hospital, Harvard Medical SchoolBoston, MA, USA; ^4^Department of Pathology, Brigham and Women’s Hospital, Harvard Medical SchoolBoston, MA, USA; ^5^Medical and Population Genetics Program, The Broad Institute of M.I.T. and HarvardCambridge, MA, USA

**Keywords:** non-coding RNA, epigenetics, gene regulation, neurodevelopment

## Abstract

Long non-coding (lnc) RNAs are defined as non-protein coding RNAs distinct from housekeeping RNAs such as tRNAs, rRNAs, and snRNAs, and independent from small RNAs with specific molecular processing machinery such as micro- or piwi-RNAs. Recent studies of lncRNAs across different species have revealed a diverse population of RNA molecules of differing size and function. RNA sequencing studies suggest transcription throughout the genome, so there is a need to understand how sequence relates to functional and structural relationships amongst RNA molecules. Our synthesis of recent studies suggests that neither size, presence of a poly-A tail, splicing, direction of transcription, nor strand specificity are of importance to lncRNA function. Rather, relative genomic position in relation to a target is fundamentally important. In this review, we describe issues of key importance in functional assessment of lncRNA and how this might apply to lncRNAs important in neurodevelopment.

## THERE IS A WIDE VARIETY OF NON-CODING RNA IN MANY SPECIES

The co-occurrence of massively parallel sequencing technology applied to RNA and the recognition that non-coding, functional RNA species may not be restricted to X-chromosome inactivation (Jeon et al., 2012; Batista and Chang, 2013) or to protein synthesis machinery, have revealed an RNA universe of remarkable diversity in plant and animal cells. Non-coding (nc) RNAs, those RNA molecules that are not templates for protein synthesis, make up a large portion of the total RNA in the cell suggesting a profound functional importance. Despite their abundance, few ncRNAs have been studied and even fewer have been functionally characterized. These ncRNAs come in many forms: they can be very small or several hundred kilobases long; they may be spliced or unspliced; they can form linear or tertiary structures; they may or may not have a poly-A tail, and some interact with DNA, protein, or other RNA molecules (Novikova et al., 2013). As is described in this review, among various roles, Long non-coding (lnc) RNAs participate in guidance of large protein complexes to DNA leading to influence over locus-specific gene expression, and in the modification of expression or abundance of complementary messenger RNA strands. The wide diversity of function and form of ncRNA, combined with the explosive growth in newly identified ncRNA molecules, has lead to a need to understand better potential relationships of function between ncRNA and to consider a more categorical approach to classification.

Non-protein coding RNA has long been recognized in cells. Transfer RNAs and ribosomal RNAs were identified over 50 years ago; neither encodes a peptide chain, though they are integral components of the machinery for protein synthesis. Identification of these RNAs demonstrated that ncRNAs interact with proteins, perform specific cell functions, and operate autonomously from information transfer. Francis Crick’s central dogma (1955) described information transfer amongst DNA, mRNA, and protein, and even at that time was recognized as an oversimplification. Crick himself subsequently built substantial flexibility into the model in 1970 such as the idea that RNA may be prone to “special” and “unknown” transfers of information (Crick, 1970). While he may not have imagined the diversity of RNA (Nakamura et al., 1996), there was a tacit acknowledgment that there was likely more to RNA than was known. Subsequent identification of ncRNAs unrelated to protein synthesis over 25 years ago, specifically, the catalytic ribozymes that formed secondary and tertiary structures thought to be important to early life on earth, re-enforced the diversity of RNA species (Sharp, 1985; Lamond and Gibson, 1990).

Several recent papers have identified new ncRNA species of particular function, and mechanistic insight into some of these different varieties of RNA reveal overlapping features, both in plant and animal cells. This diversity of RNA has been extensively reviewed with respect to small RNA-induced silencing complex (RISC)-related RNAs (e.g., Czech and Hannon, 2011) and lncRNAs (e.g., Rinn and Chang, 2012), with particular emphasis on disease specificity (Qureshi and Mehler, 2012; Sana et al., 2012) and epigenetic function (Lee, 2012). While there are several reviews that categorically describe different studies on RNA (Esteller, 2011; Wan et al., 2011), a more critical analysis of what defines a long ncRNA is lacking and the methods used for this, as well as a synthesis of lncRNA function across cell types. The purpose of the current review is to contextualize lncRNAs more generally, and review their effects in cellular function with respect to mechanism. This information will then be used to frame some of the preliminary studies emerging from studies of neurodevelopment.

## CHARACTERIZATION OF lncRNAs

Several recent reviews have delineated ncRNA species into sub-categories based on size (less or greater than 200 bases – often used as the definition of long versus short ncRNA), position (e.g., RNA species generated from the 3′UTRs or 5′UTRs), molecular interactions (e.g., Drosha- or Dicer-dependent), and molecular function, a good example of which is competitive antisense (AS) RNA that binds to microRNA and acts as a sponge to inhibit competitively microRNA from binding to a sense mRNA transcript (Cesana et al., 2011). It is unclear whether these categories are empirically determined, or whether they will prove relevant to categorization as future ncRNAs are discovered; indeed, the identification of such a wide diversity of RNA is consistent with what might be expected from an ancient, flexible molecule, capable of forming 3D structures and interacting with DNA, protein, or other RNAs.

What makes a lncRNA a lncRNA rather than some other RNA species? Are they a functionally distinct RNA product or are they a small part of the transcriptome that has been suggested to occur from large portions of the genome, mostly from recent ENCODE data (Carninci et al., 2005; Birney et al., 2007)? Certainly, a recent report (Guttman et al., 2013) suggests that intergenic lncRNAs are indeed non-coding, an issue that has been previously determined using algorithms (Lin et al., 2011) to assess whether different combinations of potential codons are similar to any other previously identified amino acid molecule. Most studies of lncRNA also attempt to determine whether an RNA species is localized to the nucleus, usually using RNA fluorescence *in situ* hybridization (FISH). Because translation occurs in the cytoplasm this might be evidence for the lack of coding potential. This analysis is somewhat arbitrary though, because ncRNA might be identified in the nuclear, chromatin, or cytoplasmic fraction of cells. Compartmentalization of lncRNAs in one of these fractions may be a defining feature of different lncRNA and may help to guide future classification schemes. Functional studies of lncRNA have also led to a proliferation of potential future categories for lncRNA, some of which are listed in **Table 1**, but this categorization creates its own problems in that many lncRNAs have overlapping features. This is a major issue at the moment and one likely to increase in complexity given the number of RNAs that can be detected from so many regions of the genome.

**Table 1:** Some examples of categorization of non-coding RNA.

Category	Description	Example	Reference
Intronic	Expressed from the intron of target	DMD lncRNA	Bovolenta etal. (2012)
H3K4me3	Has a methylated H3K4 promoter	lincRNA-p21	Huarte etal. (2010)
Antisense	Expressed from the non-coding strand and acts on the complementary target	BACE1-AS	Faghihi etal. (2008)
Enhancer	Expressed to enhance expression at a locus at some distance from target	p53 eRNAs	Melo etal. (2013)
Promoter	Acting on and expressed from the promoter of target	DBE-T	Cabianca etal. (2012)
Intergenic	Expressed at some distance from coding genes	lincRNA00299	Talkowski etal. (2012)
*Trans*-acting	Acting at some distance from target	Evf2	Bond etal. (2009)
*Cis*-acting	Acting on an adjacent target	AIRN	Santoro etal. (2013)
Small	Less than 200 bp in size	microRNA 137	Ripke et al. (2011)
Long	Greater than 200 bp in size	Fendrr	Grote etal. (2013)
5-UTR	Expressed near the 5′UTR of target	5′UTR ELK-1	Rahim etal. (2012)

The current classification system will likely evolve as more RNA species are discovered, and classification of each ncRNA might follow a similar trajectory to that of protein coding gene classification. Genes that lead to an mRNA product are not divided up by length, genomic position, whether they are spliced or not for example, and numerous coding genes fit into different classification categories. Instead they are classified by function or conserved domains. Likely it is the novelty of the RNA field, facilitated by the detection of so many transcripts by massively parallel sequencing that is leading to the classification conundrum, but this may diminish as individual RNAs are functionally analyzed.

Several recent reports have carefully documented lncRNAs over a very unique range of function. To understand how lncRNAs are similar or different in both structure and function, we synthesize this information from recent papers to determine if there are any patterns or consistencies across RNA species. We focus on currently defined long RNA (>200 bp) and omit discussion of small RNAs such as microRNA, piwiRNA, or imprinting-related RNA’s.

## FUNCTIONAL STUDIES OF lncRNAs

The recognition of *HOTAIR* (Rinn et al., 2007) as a lncRNA that regulates gene expression in* cis* and *trans *(it is transcribed on chromosome 12 from the *HoxC* cluster and can regulate the chromosome 2 *HoxD* gene cluster) opened a new chapter for RNA molecules. *HOTAIR* defined a class of molecules distinct from housekeeping RNAs, microRNAs, and others, and which were not involved in fundamental imprinting processes. It hinted at the existence of RNA in the genome with regulatory functions directly related to their particular sequence and provided an explanation for the targeting specificity required by ubiquitous binding molecules such as large chromatin modifying complexes. Since *HOTAIR*’s description, the function of many other lncRNAs has been revealed. Shown below is the large diversity of these molecules, from their genomic position in relation to the genes they regulate, their size, processing, and mechanism of action. While this diversity is large, there are also similarities, especially in reference to function. To demonstrate differences and similarities, we have selected all reports from the last 2 years (2011–2013) that have characterized positional, processing, and functional information of specific lncRNAs. **Table 2** lists structural information from lncRNAs that have been characterized functionally and this information is synthesized with the functional characteristics in the concluding remarks.

**Table 2:** Processing and positional diversity of lncRNA (in order described in text).

lncRNA [Ref]	Species	Description of all structural properties reported
COLDAIR (Heo and Sung, 2011)	*A. thaliana*	1100 base RNA expressed from the sense strand relative to target, has no poly-A tail, expressed from an intron of the target gene
IRT1 (van Werven etal., 2012)	*S. cerevisiae*	Expressed from promoter of target, 1.4 kb in length, not spliced and is transcribed from the same strand as the gene it regulates
NeST (Gomez etal., 2013)	*M. musculus*	Encoded on the antisense strand, contains six exons spread over a 45 kb region, transcript is 914 bases
Braveheart (Klattenhoff etal., 2013)	*M. musculus*	590 base RNA with three exons, 33% confined to the nucleus
NeST (Gomez etal., 2013)	*M. musculus*	Encoded on antisense strand, contains six exons spread over a 45 kb region, primary transcript is 914 bases
DBE-T (Cabianca etal., 2012)	*H. sapiens*	9.5 kb is one major product, transcribed from same strand as target genes, transcript contains one of many targets, nuclear and chromatin associated
HOTTIP (Wang etal., 2011)	*H. sapiens*	3,764-nucleotide, spliced and polyadenylated intergenic RNA, ~330 base product, regulates gene cluster
ANRIL (Wan etal., 2013)	*H. sapiens*	~126 kb transcript, spliced, 19 exons with an ~1.1 kb transcript, 13 isoforms transcribed in the antisense orientation of gene cluster, overlaps one target gene
lincMD1 (Cesana etal., 2011)	*M. musculus*	Three exons and two introns in 14 kb of genomic space, spliced product of 521 bases, accumulates as cytoplasmic poly-A+ RNA, transcribed on same strand in same orientation as microRNAs for which it acts as a decoy
TINCR (Kretz etal., 2013)	*H. sapiens*	Three exons, 3.7 kb transcript predominantly cytoplasmically expressed, over 100 different targets dispersed through genome
UCHL1-AS (Carrieri etal., 2012)	*M. musculus*	Four exons spanning 70 kb, overlaps the first 73 bases of *UCHL1*, including the AUG start codon, transcribed in reverse orientation in a head-to-head fashion, second intron of *UCHL1* contains the TSS for* UCHL1-AS*, enriched in the nucleus
1/2sbsRNA1 (Gong and Maquat, 2011)	*H. sapiens*	Present in cytoplasm, poly-A+, two alternative transcripts consist of 688 nucleotides, multiple targets throughout genome

The lncRNA *COLDAIR* presents a series of themes for lncRNAs with respect to function. *COLDAIR* recruits polycomb repressive complex 2 (PRC2), a complex of proteins that can alter histone chemical groups to decrease gene expression, through an intermediate protein (homolog of Enhancer of zeste, *Drosophila*) and the binding of *COLDAIR* occurs through a CXC domain of this intermediate protein (see **Table 3** for a discussion of RNA:protein interaction domains). *COLDAIR* is expressed at equal ratios over time, despite an increasing repression of the target, suggesting increased affinity for the PRC2 interaction. *COLDAIR* reveals several potential areas of diversity/similarity amongst lncRNAs. What determines expression of the lncRNA itself? Is the lncRNA regulated in conjunction with the target or independently from it? Is the lncRNA action direct on the target or indirect? Is the lncRNA repressive or activating? Does it act on a single target or a cluster of targets at a locus?

**Table 3:** Protein:RNA interacting domains.

**Cysteine domains**
CXC (redox-like), CXXC (redox), or C-X(6)-X (zinc finger or ring finger) motifs refer to the cysteine residue (C) with any amino acid (X) in between. These Cys residues may be active, meaning they can use their highly active sulfhydryl (SH) group to form a covalent bond with the OH group on the RNA sugar ring. These motifs can also interact with Ser, Thr, or Tyr amino acid residues to form S–S or S–O bonds on other proteins. An example of the cysteine RNA interacting domain are the Enhancer of zeste-related proteins with conserved X(6)-C-X(3)-C-X-C motifs.
**WD domains**
WD domains refer to peptide domains with rich repeats of tryptophan (W; hydrophobic) and aspartic acid (D; negatively charged) that are present in a large range of proteins. WD domains are non-catalytic and are thought to form a platform for the interaction of different cellular partners.

The lncRNA *IRT1* differs significantly from the mechanistic action of *COLDAIR*, but also functions in a repressive manner to block expression of the target gene *IME1*. *IRT1* can respond within hours to a cell stressor to aid in the inhibition of gametogenesis, which means the repressive mechanism used by IRT1 may be specific to fast-acting effects. *IRT1* completely covers the 2 kb promoter of the target gene and functions to block transcription factors from binding and promoting transcription and acts in *cis*, similar to *COLDAIR.* Because* IRT1* is transcribed over the promoter of the target gene in the sense direction, it has an identical specificity to the DNA of the *IME1* promoter. The blocking of transcription factors in combination with aiding in the establishment of a repressive chromatin state through histone methyltransferases and deacetylases suggests that IRT1 can physically hinder TFs but also guide repressive chromatin complexes. Here the repressive effects are different than COLDAIR in that repression is due to the deposition of H3K4m2 and H3K36me by factors traveling with the RNA polymerase transcribing IRT1. Little is known about the regulation of IRT1, but it must be under tight control to hinder or allow expression of the target gene within such a timeframe of only hours.

NeST is a lncRNA that functions to increase transcription of the target gene and appears to act in trans although it is physically proximal to its target gene, *Ifng*. Evidence for trans action comes from NeST being genetically unlinked to its target gene and from experimental injection of NeST into cells. NeST action on the target gene is similar to IRT1 and COLDAIR in that it acts through a histone complex, but in this case it physically interacts with WDR5, which has a WD repeat domain of ~40 amino acids (see **Table 3**). WDR5 is a core subunit of complexes that catalyze the methylation of histone H3 at lysine 4, a mark of active gene expression, so NeST interacts directly with the histone modifying complex, unlike COLDAIR. It is likely that NeST functions to physically bring the histone modifying complex in close proximity to the target gene; it is 59 kb downstream from its target in mouse and 166 kb in human.

*Bvht* is a *cis*-acting lncRNA expressed only in mouse, meaning it may be a lncRNA that has recently gained a function. In a continuing theme for lncRNAs, it interacts directly with SUZ12 (a component of the PRC2 complex) and functions upstream of a key gene in lineage commitment. This differs from the action of *COLDAIR* that requires a binding partner for interaction with PRC2, whereas *bvht* directly interacts with one of the subunits. Notably, *SUZ12* has a zinc finger motif, which may explain the protein/RNA binding (see **Table 3**).

DBE-T is a human lncRNA expressed only in a diseased condition only that acts in *cis *and affects genes in a large chromosomal region, in contrast to COLDAIR, IRT1, NeST, or bvht, which appear to regulate a single target, although these targets often trigger expression of many other genes. DBE-T is transcribed from the first repeat of the D4Z4 repeat domain that is important for recruitment of PRC2. Repression of the region, controlled by PRC2 binding and spreading, commences at the repeat region, thus the basal state in adult cells is the repression of genes at this chromosomal locus. Loss of PRC2 at the repeat region corresponds with the binding of ASHL1, a histone lysine N-methyltransferase that is part of the TrixG group, which recruits DBE-T to chromatin. Thus, this lncRNA is at the crossroads of crosstalk between conflicting histone modifying complexes. While little is known about the regulation of lncRNAs, DBE-T may be an example of a positive feedback loop which may be a common theme for other lncRNAs – in other words, lncRNA expression may be regulated by targets of the target that the lncRNA itself regulates.

Similar to the positive feedback observed between DBE-T and ASHL1, HOTTIP lncRNA and WDR5 operate analogously. Similar to NeST, HOTTIP physically interacts with WDR5, and WDR5 forms a complex with MLL1, which is a H3K4 methyltransferase, triggering gene expression. HOTTIP maintains an appropriate level of the WDR5/MLL1 at a gene cluster, and its influence over the gene cluster dissipates as a function of distance from its site of transcription. Thus, this lncRNA interacts indirectly with a histone modifying complex, is involved in a feedback loop with its interacting partner, and activates expression of a cluster of genes as a function of distance from its site of expression.

ANRIL is a lncRNA transcribed immediately upstream of a cluster of genes important in human cell proliferation and is probably the most studied lncRNA to date because of its important role in cancer. ANRIL is transcribed on the AS strand of three intimately linked genes. It can bind to the transcript of the nearest gene at the locus, *INK4*, through complementary base pairing and can act at the promoter to recruit both PRC1 and PRC2 to repress transcription. ANRIL, while seemingly with a wider diversity of function than other polycomb recruitment lncRNAs, may actually foreshadow the function of other PRC-recruiting lncRNAs. Specifically, that they may have a wide variety of functions at a particular locus, and the only reason this has not yet been identified is because of experimental design strategies. We suspect many PRC-interacting lncRNAs will have many other functions that complement their effects. The multi-mechanistic function of ANRIL also showcases the idea that not all lncRNAs operate by recruiting large histone modifying complexes. Instead, recently identified lncRNA often operate by binding to the primary target or acting as a decoy of repressive effectors of the target.

lincMD1 and TINCR are two examples of non-PRC-recruiting lncRNAs with novel function to refine expression of a target. In contrast to lncRNAs COLDAIR, IRT1, NeST, BVHT, DBE-T, HOTTIP, and ANRIL, lincMD1, is a lncRNA that appears to be a by-product or remnant of microRNA processing (Ala et al., 2013). Specifically, this lncRNA can act as a decoy for the targets of microRNA produced from the same locus as lincMD1 (Cesana et al., 2011). TINCR also differs from all reported lncRNAs to date as it appears to bind to a 25 bp “TINCR-box” present in the RNA of different coding transcripts and influence levels of these transcripts in a STAU-dependent manner. STAU is an RNA guidance protein initially identified for its involvement in oocytes of *Drosophila*. All lncRNA described to date provide locus specificity for activating or repressive complexes to neighboring target genes, or interact directly with a target through sequence complementarity. TINCR on the other hand, appears to target specific RNA transcripts actively through an RNA sequence motif. lincMD1 also diverges drastically in that it is a by-product of pri-microRNA processing and acts to sponge the microRNAs from which it was initially processed. There may be many other pri-microRNA by-products that function similarly.

**FIGURE 1:**
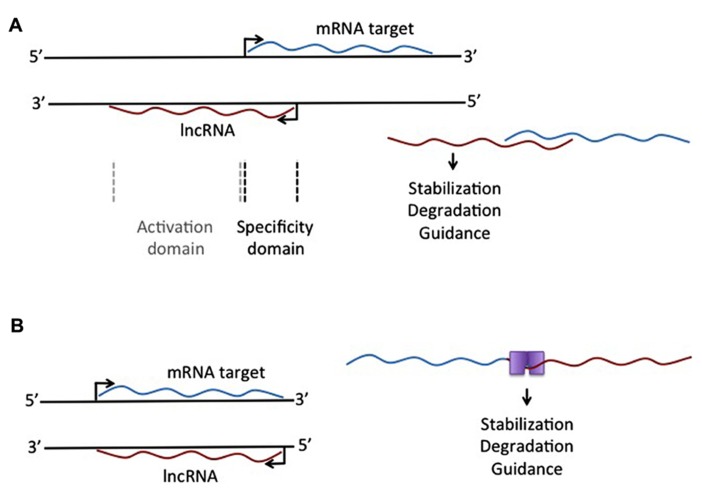
**Different mechanistic action of lncRNAs that overlap an mRNA target. (A)** lncRNA expressed from the antisense strand may have an overlapping domain, providing specificity, and a non-overlapping activation domain. This activation domain could be a sequence that allows single-strand binding for different molecules, leading to stabilization or degradation of the RNA:RNA complex. **(B)** Complementary binding of overlapping mRNA and lncRNA could create a double-stranded binding site for protein binding, leading to selective degradation of stabilization of the RNA:RNA complex.

Another lncRNA that reportedly does not use large histone modifying complexes to alter a target, but instead operates through binding of a primary target, is AS-UCHL1. AS-UCHL1 has been shown recently to be important for proper targeting of sense transcript to polysomes, suggesting a stabilizing function for this lncRNA, demonstrated by a strong increase in UCHL1 protein with no difference in UCHL1 transcript on over-expression of AS-UCHL1. This principle of RNA stabilization to affect protein levels of targets may be a continuing theme for lncRNAs (e.g., Yoon et al., 2012). This lncRNA has a single target, binds it directly, and functions to increase protein of the primary target by stabilizing the mRNA. Besides this novel functional effect for a lncRNA, AS-UCHL1 action is driven by repeat elements within the AS transcript. Specifically, an orientation-specific SINEB2 repeat is required for the stabilizing function and protein synthesis activation of the sense strand. The overlapping portion of the AS gene with the sense gene thereby provides targeting information, while the SINEB2 region, which is not overlapped by the sense strand, confers protein synthesis activation (see **Figure 1A**).

The idea of repeat elements in the genome, acting through lncRNAs, has also been described with respect to Alu repeats, one of the most common repeats in the human genome. The description of overlapping Alu repeats, one in an AS strand and one in the 3′UTR of the sense strand, can lead to formation of a STAU1 binding site, which allows for STAU1 to stabilize base pairing and target the RNA duplex for degradation. Similar to all lncRNAs described here, these Alu-containing lncRNAs can regulate the levels of a transcript through an mRNA decay pathway. This was specifically demonstrated for *SERPINE1* and *FLJ21870* mRNAs between their 3′ UTR Alu element and the Alu element in a single lncRNA (see **Figure 1B**).

## SYNTHESIS OF lncRNA FEATURES FROM DIFFERENT SPECIES

These examples support an important role for lncRNAs in the genome, and highlight the diverse function of lncRNAs, but also some similarities. First, there appears to be no relationship between the particular function of a lncRNA, its size, or how it is processed. This suggests that lncRNAs will represent a diverse range of characteristics. Second, neither transcriptional direction nor strand specificity appears to have an effect on function. The key element is that lncRNAs are produced either within their target gene or in the vicinity of target genes. Those lncRNAs produced from overlapping regions of their target gene are more likely to bind to the target, however, due to direct complementarity with the target. Whether these lncRNAs come from the same or the AS strand as a target appears not to have functional impact. Future experiments should document this for all newly described lncRNAs to determine whether this remains the case. These ideas may help guide issues of categorization of lncRNAs, and we propose a system that anchors lncRNAs in the target molecule. This may not prove useful for those lncRNA, like the intergenic lncRNAs, that do not appear to have nearby targets. Their function may prove to be completely different and independent from those lncRNAs expressed in relationship to mRNAs.

Most lncRNAs are modulators of a primary transcript suggesting that, evolutionarily, they arose after the primary transcript. For example, HOTTIP, either evolved with or after the *HOX* gene cluster that it regulates. There is little evidence for lncRNAs that operate in isolation (although the lincRNAs may be an exception, reflected by their distinct locations and conservation across species (Managadze et al., 2013), but rather form part of a transcriptional regulation complex of a specific target or a cluster of targets. This suggests that characterizing lncRNA might best be done grounded in the primary target rather than through effector status.

Many lncRNAs act though histone modifying complexes and appear to affect either a single target gene or a cluster of genes in a local region. They may require an intermediate binding partner for recruitment of the histone complex or interact directly with one of the proteins in these complexes. Determining whether lncRNAs bind directly to the target, interact directly with a histone modifying complex, or require a partner to bind histone modifying complex, will be important information as new lncRNAs are uncovered. Most lncRNAs do not share any sequence similarity (i.e., no indication yet of any conserved domain within lncRNAs) and it seems the position of lncRNAs in relation to the target(s) are of fundamental importance to their function. While there are many remarkable functions attributed to lncRNAs, we strongly suspect that the function of even these lncRNA will prove more diverse as they undergo further investigation.

## lncRNAs IN CNS DEVELOPMENT

Functional and mechanistic data generated by studying ncRNAs in different molecular systems and species suggests lncRNAs likely play an important role in all cellular systems. As evidenced in the previous sections, lncRNAs most likely act as modifiers of a complementary RNA, interact with large histone complexes, interact with complementary DNA sequences, or act completely independently in the nucleus with no obvious partners required. Given this diverse potential, the complexity of the nervous system in any species might be partially due to the additional level of control over the cellular machinery by lncRNAs. lncRNAs may provide a means to tweak a cellular system at many levels and to operate rapidly in response to external signals whether axon guidance cues or environmental exposure. In line with these ideas, we synthesize recent reports of lncRNAs in the developing nervous system.

## lncRNA IN NEURAL STEM CELLS

Some of the first experiments to underscore the importance of lncRNAs were done in mouse or human stem cells from. Stem cells used for research are either derived from the inner cell mass of a fertilized embryo (Thomson et al., 1998) or induced to pluripotency by the experimental increase of transcription factors normally present in early embryonic stages (Takahashi and Yamanaka, 2006) in terminally differentiated cells. These stem cells can be differentiated to a neural stem cell (NSC) fate and these NSCs can then give way to glia and neurons (Hu et al., 2010). In a wide ranging, exploratory analysis, Ng and colleagues (Ng et al., 2012) examined neuronal differentiation from human embryonic stem cells (hESCs). They used a two-step differentiation protocol from radial glial-like cells to largely dopaminergic cells, and then assessed global gene expression levels of pre-selected lncRNAs in radial-glial cells compared to dopaminergic-like cells. They identified 35 lncRNAs that were differentially expressed between progenitor and mature states, and then tested some of these for functionality. Following similar designs of non-neuronal studies of lncRNA, they assessed the association of differentially expressed lncRNA with SUZ12 and the neurogenesis repressor complex REST/NRSF (neural restrictive silencer factor; Naruse et al., 1999). In a study using just three lncRNAs, their data supported interaction of one lncRNA with REST and another lncRNA with SUZ12. While the SUZ12 interaction is consistent with previous lncRNA studies, the interaction with REST/NRSF is novel for lncRNAs in neurons, although it does associate with HOTAIR in non-neuronal cell types to repress expression of neuronal genes. This suggests that the lncRNA in the Ng et al. (2012) study may interact with REST to regulate neuronal gene expression.

While ES (embryonic stem cells)- and iPSC (induced pluripotent stem cell)-derived NSCs may not perfectly capture the developmental progression of the human brain, they provide an excellent model with which to screen for important factors as the cells develop from stem cells to electrically active neurons. A study monitoring iPSC-NSC differentiation accompanied by RNA sampling at different timepoints, contrasted with brain temporal lobe brain tissue RNA levels from the same donor has revealed a gradual increase in the expression of different lncRNAs as NSCs differentiate (Hjelm et al., 2013). This is supported by our own study, where we observed an increase in the neurodevelopmentally important intergenic lncRNA00299 as iPSC-NSCs differentiated (Talkowski et al., 2012). A recent report using adult NSCs in mice has further confirmed that lncRNAs increase as cells differentiate. Ramos et al. (2013) sorted stem cells of the sub-ventricular zone of mice and screened these cells for expression levels of different lncRNAs creating publically accessible expression maps for lncRNAs that may be relevant to glial-neuron specification in adult brain.

## lncRNA IN DEVELOPING BRAIN

In the mammalian brain, lncRNAs have long been recognized as important in neurodevelopment, although they were traditionally referred to as AS transcripts. An example of this is the AS transcripts near the *Sox4* and *Sox11* loci produced during development of the mouse cerebral cortex (Ling et al., 2009). Sox proteins contain a high mobility group, and this refers to the ability of these proteins to bind and bend DNA. Using global gene expression analysis tools, Ling et al. (2011) showed that AS *Sox4* and *Sox11* transcripts are produced during proliferating and differentiating states, suggesting that the regulation of these important genes is by complementary lncRNAs. Recently, this same group documented a similar effect with respect to *Nrgn* and *Camk2n1* gene product in mouse cerebral corticogenesis. A recent study in adult brain also suggests that electrical activity in neurons stimulates lncRNA (Barry et al., 2013). Given the importance of initial synaptic contacts and communication between cells, it stands to reason that there may be lncRNAs that respond to activity and independent lncRNAs that increase expression as differentiation proceeds, similar to data from *in vitro* NSC models. Of intense interest as well are the loci of the genome where AS transcripts are transcribed from the same genomic locations as brain-relevant genes and how some of these may be specific to human. For example, *BDNF* is transcribed from chromosome 11 and an AS transcript is produced from the opposite strand in humans but not in mouse (Aid et al., 2007; Pruunsild et al., 2007). The discovery of an ever increasing number of AS transcripts that may assist in regulation of genes fundamental to brain development will likely be forthcoming. Determining the exact role of these AS transcripts, their size, and binding dynamics will be important.

Recent data from our group suggest that lncRNAs may be important in neurodevelopmental disease (Talkowski et al., 2012); we showed that a nuclear, multi-exon lncRNA was disrupted in subjects with global developmental delays. This complements work from others, where ncRNAs have long been suspected of causing certain neurological problems, the best example of which may be Prader–Willi syndrome (PWS). PWS is characterized by intellectual disability, sleep disorders, and psychosis, and can be caused by deletion of 15q11-13 on the paternal chromosome. Genes in this region are suppressed on the maternal chromosome, meaning that paternally expressed genes likely provide the optimal dosage of expression. The minimal required locus within this ~10 Mb region implicates *116HG*, a lncRNA retained in the nucleus, as well as the small nucleolar RNA *SNORD116 *(Sahoo et al., 2008). Both ncRNAs are the control of the imprinting control region, involving multiple overlap of genes – suggesting that transcription and splicing in this region are complex. Recently, Powell et al. (2013) reported the first experiments to determine the function of lncRNA 116HG. They found that 116HG forms RNA “clouds” specific to nuclei in mouse brain, and that these 116HG clouds change size and shape in predictable ways as the brain develops. Using RNA and DNA FISH mapping, they show that 116HG likely interacts with the paternal *UBE3a* locus, a gene found immediately upstream of the 116HG locus and known to be important in neurodevelopment. Their data further suggest that 116HG interacts with RBBP5, a subunit of the MLL complex, which acts as a transcriptional activator by methylation of H3K4. This model conforms nicely to what is known of lncRNA functions in other species; 116HG might associate with MLL complex and interact with histones at the *UBE3a* locus. How 116HG itself is regulated is unknown, but this will be clearly important to understand better the neurobiology of PWS.

lncRNAs likely have a role in many aspects of the cell, and brain development might be an area where their structure and function is particularly suited. This may suggest that many more lncRNAs await discovery in novel systems as well as in added layers of control for well known processes of neurodevelopment.

## Conflict of Interest Statement

The authors declare that the research was conducted in the absence of any commercial or financial relationships that could be construed as a potential conflict of interest.
